# Dopaminergic and serotonergic genetic variants predict actions and expectations of cooperation and punishment

**DOI:** 10.1038/s41598-025-03772-4

**Published:** 2025-07-01

**Authors:** Pablo Marcos-Prieto, Erica Ordali, Veronica Mariotti, Sara Palumbo, Stefano Vellucci, Emiliano Ricciardi, Leonardo Boncinelli, Pietro Pietrini, Silvia Pellegrini, Ennio Bilancini

**Affiliations:** 1 IMT School for Advanced Studies Lucca, P.zza San Francesco 19, 55100 Lucca, Italy; 2 Department of Economics and Management, University of Firenze, Via Delle Pandette 32, 50127 Firenze, Italy; 3 Department of Clinical and Experimental Medicine, University of Pisa, Via Roma 67, 56126 Pisa, Italy

**Keywords:** Genes, Dopamine, Serotonin, Cooperation, Public goods game, Punishment, Human behaviour, Behavioural genetics

## Abstract

Genetic variants in dopaminergic and serotonergic pathways have been linked to individual differences in social behavior. In this study, we investigated the relationship between eight allelic variants within these pathways and both behavior and beliefs in 99 participants playing an online Public Goods Game (PGG) with and without punishment. Our results show that individuals with the 5-HTTLPR L/L genotype contributed less and had lower expectations of others’ contributions in the absence of punishment; the 5-HTR1B-rs13212041 T/T genotype was associated with lower expectations of antisocial and spiteful punishment; the COMT-rs4680 A/A (Met/Met) genotype was linked to lower expectations of contributions in the presence of punishment. These findings suggest that specific alleles modulate both cooperative behavior and social expectations, suggesting a genetic contribution to individual variability in responses to social dilemmas.

The evolution of human cooperation understood as the propensity to incur personal costs to benefit another individual or group, is a scientific dilemma that has consistently attracted interest over the decades. In traditional Game Theory and Economics, cooperation in one-shot interactions is not an optimal strategy, whereas being a “defector” allows one to maximize personal benefit by acting selfishly. However, multiple experimental studies have consistently shown across a wide range of contexts that humans tend to cooperate—and do so for different reasons^1^. Several mechanisms have been identified that support the selection of cooperation, including, among others^2^, peer punishment of defectors, also known as prosocial or altruistic punishment^3^. Prosocial punishment involves paying a cost to reduce the profits of defectors or free riders, i.e., those who do not cooperate but still benefit from others’ cooperation. However, punishment might be directed not only toward defectors but also toward cooperators, which has been labeled antisocial punishment. Given that theoretical models predict the instability of cooperation in the presence of antisocial punishment^4–6^, and that antisocial punishment has been observed in social dilemmas^7,8^, the cooperation puzzle remains far from being solved.

Neurotransmitters such as serotonin and dopamine play roles in a variety of cognitive and affective processes, including mood regulation, pleasure, and motivation^9^. Moreover, they contribute to sensory inference and decision-making^10^. Selective modulation of these neurotransmitters has proven to be associated with several aspects of social behavior. For example, serotonin depletion induced by a tryptophan-free diet has been shown to increase altruistic punishment^11^ and reduce acceptance of unfair offers in the Ultimatum Game^12^, as well as to reduce the level of cooperation in the Prisoner’s Dilemma^13^. On the contrary, the potentiation of serotonergic neurotransmission by the serotonin reuptake inhibitor citalopram made subjects more likely to judge as emotionally salient, morally unacceptable harmful actions, suggesting that serotonin might promote prosocial behavior by enhancing harm aversion^14^.

Concerning dopaminergic neurons, they are believed to play a central role in economic decision-making by reinforcing actions that yield outcomes exceeding expectations, a mechanism driven by the reward prediction error process^15^. Moreover, pharmacological increases in dopamine availability in individuals treated with the dopamine precursor levodopa have been associated with reduced altruism in decision-making contexts where financial gain involves inflicting pain on oneself or others^16^. Furthermore, optogenetic activation of dopaminergic neurons in the ventral tegmental area (VTA) has been shown to drive aggression induced by social isolation^17^. Finally, D2 receptor antagonists are commonly used to pharmacologically manage pathological aggression, supporting the notion that heightened dopaminergic neurotransmission plays a causal role in aggressive behavior^18^.

Overall, these findings suggest distinct roles for serotonin and dopamine in shaping prosocial and antisocial behaviors, highlighting polymorphisms in serotonergic and dopaminergic pathways as promising candidates for investigating individual differences in various aspects of human sociality, including cooperation and punishment. The literature exploring whether—and to what extent—genes influence humans’ propensity for cooperation or aggression is extensive and methodologically diverse, with several candidate genes identified as being associated with cooperative behaviors, antisocial-like behaviors, or both^19^.

For example, in the promoter region of the SLC6A4 gene that encodes for the serotonin transporter (5-HTT), which controls the strength and duration of serotonergic neurotransmission by regulating the serotonin reuptake from the synaptic cleft to the presynaptic terminal of serotonergic neurons, is located a functional polymorphism known as 5-HTT Linked Polymorphic Region (5-HTTLPR)^20^. 5-HTTLPR is a variable number of tandem repeats (VNTR) consisting of 20–23 base pair sequences, repeated 13 to 22 times. The two more frequent alleles are a short one (S), with 14 repeats, and a long allele (L), with 16 repeats. The L allele, as compared to the S allele, is responsible for an increase in gene expression leading to a more rapid 5-HT reuptake^21,22^. Homozygotes for the L allele tend to be less anxious, more prone to risky behavior, and appear more sensitive to unfairness^23^ than S allele carriers. Existing literature has reported associations between the 5-HTTLPR S allele and various aspects of antisocial behavior, including aggression, violence, criminality, and hostility. However, only a few studies have reported similar effects for the L allele or found no significant impact^24^. Furthermore, L/L individuals have been shown to rate “unintentional harm” in moral dilemmas as more acceptable compared to S allele carriers^25^. Additionally, they have been described as having heightened sensitivity to punishment and social norms^26^.

Among serotonin receptors, the postsynaptic 5-HTR2A and the presynaptic 5- HTR1B have been the most extensively studied concerning social behavior. The 5-HT2A receptor, one of the primary excitatory serotonin receptor subtypes among G protein-coupled receptors, is involved in the effects of many antipsychotic drugs^27^. According to Schroeder et al.^26^, the 5-HTR2A allelic variants appear to be implicated in punishment sensitivity, resulting in more cooperative behavior under the threat of punishment. Moreover, rs6311 (C/T, in exon 1), rs6313 (T102C, in exon 1), rs6314 (C1354T, His452Tyr, in exon 3), and rs7322437 (A/T, intronic) have been associated with psychiatric disorders, hostility, and aggression^28,29^. Notably, the rs6314 variant has been linked to changes in 5-HTR2A expression in the brain, cognitive function, and treatment response^30^. The 5-HTR1B autoreceptors exert an inhibitory control on extracellular serotonin levels through a negative feedback mechanism by increasing 5-HTT-mediated serotonin reuptake^20,31^. Different polymorphisms of the 5-HTR1B gene (e.g., rs6256, rs130058, and rs13212041) have been found associated with impulsivity and antisocial behavior in humans^32,33^. In particular, rs13212041 (T/C), located in the 3’untranslated region of 5-HTR1B gene, enables a microRNA interaction that reduces gene expression only when the T allele is present, not the C allele^32^. The low-expression T allele has been associated with greater anger, hostility, and psychopathic traits in men compared to high-expression haplotypes^33,34^.

Serotonin levels are also influenced by its synthesis rate, which is dependent on the activity of tryptophan hydroxylase. The tryptophan hydroxylase-2 (TPH2) is a brain-specific isoform of the rate-limiting enzyme in the biosynthesis of neuronal serotonin. A single-nucleotide polymorphism (SNP; rs4570625 G-703 T) in the promoter region of TPH2 gene, is associated with emotion recognition and cognitive functions. Carriers of T-allele (T/T or T/G), as compared to the G/G carriers, exhibit greater amygdala reactivity to facial emotions^35^, greater prefrontal areas activation during working memory tasks^36^ and poorer cognitive control in cognitive tasks^37^. A more recent study has shown a significant association between the TPH2 rs4570625 T/T genotype and lower levels of impulsivity and aggressive behavior^38^. Furthermore, this genotype has been linked to more altruistic punishing behavior in the Ultimatum Game^39^. Despite these associations, the function of this SNP remains unclear^40^. Although no direct functional effect of rs4570625 on TPH2 expression has been demonstrated, the SNP is in partial linkage disequilibrium with a haplotype block comprising four SNPs (rs2171363, rs4760815, rs7305115, and rs6582078) that are strongly correlated with TPH2 mRNA expression in the adult pons.

Regarding dopamine, the COMT gene that encodes the enzyme catechol-O-methyltransferase responsible for dopamine metabolism, has been extensively investigated in association with social behavior; it has a well-known polymorphism (rs4680), where a valine (Val) is substituted with a methionine (Met) at position 158 (Val158Met) due to a G/A change in exon 4. This substitution reduces the enzyme activity by a factor of four, resulting in a slower inactivation of catecholamines^41^. The Val allele has been associated with antisocial behavior, anger, and hostility^42,43^. The Met/Met genotype has been associated with impaired risk–benefit evaluation and underestimation of action costs^44^, as well as reduced cooperativeness in teamwork settings and lower scores on standardized measures of cooperativeness^45^. This genotype is also associated with increased activation of the nucleus accumbens in response to punishment, presumably due to elevated dopamine availability^46^.

Among dopamine receptors, the most widely studied is the dopamine receptor D4 (DRD4). The DRD4 gene contains a 48-bp variable number tandem repeat (VNTR) in exon III, coding for 16 amino acids^47^. In humans, the 4-repeat (4r) allele is the most prevalent, followed by the 7r and 2r alleles in Caucasian and Asian populations, respectively^48^. The 7r allele has been associated with reduced altruism and empathy, higher impulsivity, and higher risk for aggression^49,50^, as well as externalizing behaviors in children^51^. The 4r allele has been instead associated with altruism and fairness preferences^52–56^.

The DRD2 gene polymorphisms have also been widely investigated; for example, the G allele of rs1799978 (A/G), A allele of rs4581480 (T/C), C/C genotype of rs1079598 (T/C), A/A genotype of rs12364283 (A/G), and T/T genotype of rs1799732 (C/T) have all been linked to several aspects of antisocial behavior, in particular aggression and impulsivity^49,57^. Data concerning the functional effects of these SNPs is scarce, except for the rs1799732 for which some evidence exists—though even in this case the available data are limited^49,58^. More attention has attracted the rs1800497 (C/T) polymorphism of the Ankyrin Repeat and Kinase Domain Containing 1 (ANKK1) gene, which is located upstream and influences the expression of DRD2 gene^59^. T allele carriers have a 30–40% decrease in striatal DRD2 density, a reduction of negative feedback on dopamine release, and an increase in dopamine synthesis ^60^. The T/T genotype emerged as significantly associated with antisocial-like behaviors and disorders^57,61,62^, whereas the T allele (T/T plus C/T) has been linked more to impulsivity and novelty-seeking^61^. Moreover, the T allele has also been associated with more cognitive and less emotional moral decision-making in the context of moral dilemmas, favoring a more rationally driven decision process^63^.

Genetic variants of the dopamine transporter DAT1 have also been investigated. DAT1 is responsible for synaptic clearance of dopamine, which limits the duration and intensity of dopamine receptor activation^64^. DAT1 is encoded by the solute carrier family 6 member 3 (SLC6A3) gene, which has several SNPs in the promoter region (rs2652511 C/T, rs2975226 A/T, rs2550948 C/T, rs255093 A/C) associated with several psychiatric disorders^65,66^. DAT1 also includes a 40-bp VNTR in the 3’untranslated region associated with impulsive and externalizing behaviors. Alleles with nine repeats (9r) or ten repeats (10r) are the most common, with their frequencies varying across different ethnicity^67^. The SLC6A3 VNTR modulates the DAT1 expression, as the 9-repeat allele decreases DAT-binding capacities and increases dopamine availability^68^. The 10r allele has been associated with impulsivity, antisocial and criminal-like behaviors^69,70^. Overall, the 9r/9r genotype was shown to be protective against a spectrum of risky behaviors in comparison to the 10r allele^70^. Furthermore, the 9r allele has been associated with more cognitive moral choices^63^.

Given that these eight genes are involved in serotonergic and dopaminergic functioning are implicated in emotion regulation, impulsivity, prosociality/antisociality, and punishment behaviors, we investigated whether eight functional allelic variants—ANKK1-rs1800497, SLC6A3 VNTR, DRD4 VNTR, COMTrs4680, TPH2-rs4570625, 5-HTR1B-rs13212041, 5-HTR2A-rs6314, and 5-HTTLPR- contribute to individual differences in cooperative and punishing behavior in two versions of a one-shot Public Goods Game.

We focused on the Public Goods Game (PGG) because it embodies the fundamental tension between individual and group benefits, capturing a typical social dilemma in which individuals have no direct personal incentive to contribute. In this context, economic games from Behavioral and Experimental Economics, which can elicit relevant proxies of social behavior within controlled settings^71^, are valuable tools for Behavioral Genetics. Given the importance of peer punishment as a deterrent against free riding, its potential genetic underpinnings have been extensively studied^11,14,23,39,53^. In contrast, alternative punishment modalities—such as punishing cooperators or punishing players at random—have not yet been investigated in the context of Behavioral Genetics. For this reason, we examined the role of our selected genetic variants in modulating antisocial punishment behavior in the PGG, in a sample of 99 healthy individuals. We consider both antisocial punishers, i.e., those who punished individuals who contributed more, and spiteful punishers, i.e., those who punished individuals regardless of their contribution^7,8^. Subjects that did not punish and those who punished low contributors were considered as the reference group to be compared with the antisocial one. Furthermore, as little is known about the relationship between genetic variants and individuals’ beliefs about others’ behavior, we also examined participants’ expectations regarding contributions and punishment in the PGG, as an indicator of general social attitudes.

Based on previous findings, we hypothesized that associations between the different genotypes within each variant and cooperative and/or punishing behavior may emerge, such as lower cooperation (and more punishment) for genotypes linked to impulsive, risky, and antisocial behaviors, and greater cooperation (and less punishment) for those associated with prosocial tendencies. To the best of our knowledge, this is the first study to simultaneously investigate the associations between eight genetic polymorphisms from both the serotonergic and dopaminergic pathways and cooperative and punishing behaviors, in conjunction with participants’ expectations about these behaviors—a connection not previously explored.

## Results

**Fig. 1:**
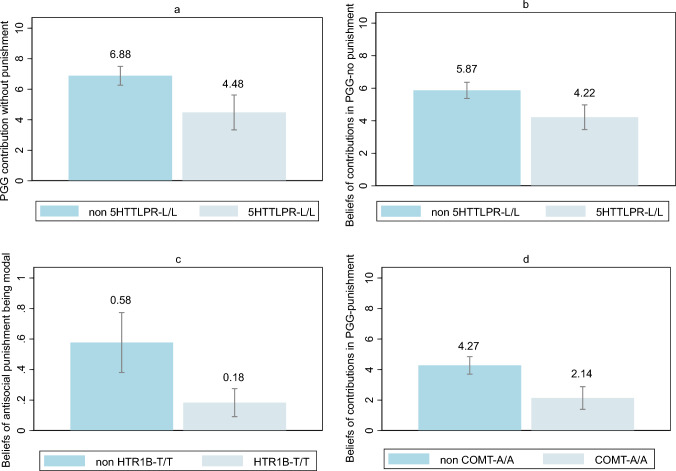
Plot representation of the four main results. Panels (**a**) and (**b**) show results for carriers vs. non-carriers of the L/L genotype of 5-HTTLPR about the average contributions to the public good in the absence of a punishment option and the average beliefs about that same behavior, respectively. Panel (**c**) shows the average beliefs about the presence of antisocial behavior for carriers vs. non-carriers of the T/T genotype of 5-HTR1B. Finally, panel (**d**) shows the average beliefs about the average contribution to the public good, in light of a punishment option, for carriers versus non-carriers of Met/Met genotype of COMT-rs4680. Bars represent standard errors.

An exploratory Spearman correlation analysis was conducted to examine the relationship between the genotypes of interests and contributions, punishment behaviors, and beliefs in the two versions of our one-shot Public Goods Game. Correlation results are shown in Table 1. A first glance at the correlation matrix indicates trends between various genotypes of both serotonin and dopamine pathways and behavior and beliefs in both versions of the Public Good Game. The results that survived Bonferroni correction were subsequently confirmed with non-parametric tests, which were also corrected. Figure 1 and Table 2 summarize our main findings.

**Table 1:** Spearman correlation matrix.

Variables	Public goods game		Public goods game with punishment			
	$PGG_{c}$	$b{\text{PGG}}_{c}$	$PGG_{p}$	$bPGG_{p}$	$ANTI$	$bmANTI$
Serotonin pathway						
HTR1B—T/T	0.1269 (0.2108)	− 0.0042 (0.9667)	− 0.2140 (0.0335)*	− 0.1634 (0.1060)	− 0.1571 (0.1205)	− 0.3850 (0.0002)***
p corrected	(1)	(1)	(1)	(1)	(1)	(0.0160)*
5HTTLPR—L/L	− 0.3553 (0.0003)***	− 0.3440 (0.0005)***	− 0.0565 (0.5786)	− 0.0377 (0.7110)	− 0.0385 (0.7051)	− 0.1820 (0.0743)
p corrected	(0.0148)*	(0.0235)*	(1)	(1)	(1)	(1)
HTR2A—T/T	0.0594 (0.5589)	0.0803 (0.4295)	0.1322 (0.1922)	0.1396 (0.1683)	− 0.0508 (0.6173)	0.1602 (0.1170)
p corrected	(1)	(1)	(1)	(1)	(1)	(1)
TPH2-T/T	− 0.0468 (0.6453)	− 0.0131 (0.8978)	0.0184 (0.8569)	− 0.0680 (0.5037)	− 0.0508 (0.6173)	− 0.0650 (0.5269)
p corrected	(1)	(1)	(1)	(1)	(1)	(1)
Dopamine pathway						
DAT-1-9r/9r	0.0006 (0.9953)	0.0985 (0.3319)	0.2942 (0.0031)**	0.2019 (0.0451)*	0.2488 (0.0130)*	0.3078 (0.0022)**
p corrected	(1)	(1)	(0.1)	(1)	(1)	(0.2)
ANKK1-T/T	− 0.1045 (0.3034)	− 0.0131 (0.8978)	0.1083 (0.2860)	0.0644 (0.5264)	− 0.0508 (0.6173)	− 0.0650 (0.5269)
p corrected	(1)	(1)	(1)	(1)	(1)	(1)
DRD4-4r/4r	− 0.1365 (0.1778)	− 0.0959 (0.3451)	0.0591 (0.5612)	0.0255 (0.8020)	0.0984 (0.3326)	− 0.0253 (0.8061)
p corrected	(1)	(1)	(1)	(1)	(1)	(1)
COMT-A/A	− 0.0403 (0.6921)	− 0.0490 (0.6303)	− 0.2733 (0.0062)**	− 0.3533 (0.0003)***	0.0336 (0.7411)	0.1820 (0.0743)
p corrected	(1)	(1)	(0.2)	(0.0160)*	(1)	(1)

The table reports the results of Spearman correlations. Uncorrected (P_U_) and Bonferroni corrected *p*-values (P_C_) are reported. Results qualitatively align when alternatively using Kendall Tau Correlation. Standard errors in parentheses, ***p < 0.001, **p < 0.01, *p < 0.05.

**Table 2:** Main findings: descriptive statistics.

Genetic variant	Behavioral variable	Effect-size	Marginal effect	[95% conf. interval]	R-squared
5HTTLPR, L/L	$bPGG_{c}$	0.8859	− 2.4033	[− 3.6913, − 1.1153]	0.1241
5HTTLPR, L/L	$PGG_{c}$	0.7571	− 1.6510	[− 2.5510, − 0.7510]	0.1005
-HTR1B, T/T	$bmANTI$	0.7817	− 0.3938	[− 0.6089, − 0.1788]	0.1482
COMT, A/A	$bPGG_{p}$	0.8437	− 2.1364	[− 3.0675, − 1.2053]	0.1240

The table reports the descriptive statistics of the four main results. Glass’$\Delta$ was used to compute effect sizes, using the standard deviations of the non-target genetic variable. Marginal effects and confidence intervals are computed through linear regression (linear probability model) with robust errors. Results qualitatively align when alternatively using Tobit (Probit) estimation.

### Effects of 5-HTTLPR gene variation on contributions and beliefs in the Public Goods Game without punishment

The correlation analysis pointed out a negative association between the L/L genotype of 5-HTTLPR and contributions in the PGG without punishment, indicating that carriers of this variant were, on average, contributing less of their endowment (r =  − 0.3553, corrected *p* = 0.0148). The result was additionally confirmed by nonparametric testing (Wilcoxon-Mann–Whitney test, corrected *p* = 0.0210). On average, a linear regression with robust errors showed that the 5-HTTLPR L/L genotype was associated with a reduction in contributions of 2.4 points, accounting for about 12% of the variance (coefficient’s corrected *p* = 0.0170) (Fig. 1a, Table 2). Additionally, the L/L genotype of 5-HTTLPR was also negatively associated with beliefs about the average contribution in the same game (r =  − 0.3440, corrected *p* < 0.0235). Carriers of the L/L genotype believed others’ contributions to be smaller, a result that was confirmed by a non-parametric test (Wilcoxon-Mann–Whitney test, corrected *p* = 0.0317). On average, the 5-HTTLPR L/L genotype decreased beliefs about others’ contributions by 1.65 points, explaining about 10% of the variance (coefficient’s corrected *p* = 0.0210) (Fig. 1b, Table 2). Overall, these findings indicated that the high expression genotype of 5-HTTLPR, which reduces serotonergic signaling, was associated with a lower contribution in the PGG without punishment and lower expectations about others’ contribution in the same setting.

### Effects of 5-HTR1B gene variation on beliefs in the Public Goods Game with punishment

Correlation analysis showed that the T/T genotype of 5-HTR1B-rs13212041 was less frequent in subjects who believed that the antisocial or spiteful punishment behavior was the most common in the group of PGG players (r =  − 0.3850, corrected *p* = 0.0160). This finding was confirmed by Pearson’s chi-squared test (corrected p = 0.0072). The marginal effect of 5-HTR1B-rs13212041 T/T genotype on beliefs, obtained by using the linear probability model with robust errors, was − 0.3938, explaining about 15% of the variance (coefficient’s correct p = 0.0216) (Fig. 1c, Table 2). Overall, these findings suggested that the 5-HTR1B-rs13212041 T/T, which increases serotonergic transmission, may predispose to more optimistic beliefs about others’ punishing attitudes.

### Effects of COMT gene variation on beliefs in the Public Goods Game with punishment

The Met/Met (A/A) genotype of COMT-rs4680 negatively correlated with the belief that others’ contributions in the PGG with punishment were smaller (r =  − 0.3533, corrected *p* = 0.0160), a result that was confirmed by non-parametric testing (Wilcoxon-Mann–Whitney test, corrected *p* = 0.0225). The variance in beliefs explained by COMT-rs4680 A/A was about 12%, with this gene variant associated with the belief that the contributions of others were lower by 2.14 points on average (coefficient’s corrected *p* < 0.001) (Fig. 1d, Table 2). Overall, carriers of the Met/Met genotype, which slows the metabolization of dopamine, appeared to have more pessimistic beliefs about the average contribution in the PGG with a punishment option.

## Discussion

This study investigated how serotonergic and dopaminergic gene variants relate to behavior and beliefs in social dilemma contexts. Using two versions of the Public Goods Game, one with and one without punishment, we found that specific alleles were associated with individual contribution levels and expectations about others’ contributions and punishment behavior. These findings support the idea that genetic factors can shape both prosocial and antisocial tendencies and individuals’ anticipations of others’ behavior. Importantly, although not all the genetic variants yielded statistically significant results, we observed non-significant trends that align with previously reported directions in the literature (see Figs. 1 to 8, in the Supplementary Material). Interestingly, since beliefs about others’ behaviors have been not previously investigated in these types of studies, our study establishes a reference point for future investigations.

In particular, our results align with previous evidence showing that reduced serotonergic signaling is linked to lower cooperative tendencies ^13^. We found that carriers of the L/L genotype of 5-HTTLPR—which is associated with increased serotonin reuptake and thus lower serotonin availability—contributed less to the public good in the absence of punishment, suggesting a tendency toward free-riding behavior (Fig. 1a). Additionally, these individuals were more likely to expect others to behave similarly, suggesting that this genotype may influence not only social behavior but also expectations (Fig. 1b). Given that the long (L) allele is associated with more efficient reuptake and consequent lower serotonin levels, individuals with the L/L genotype might be less anxious and more prone to risk-taking. Their behavior also appears to be more responsive to social norms and punishment^22,26,31^. Based on our findings, we hypothesized that carriers of the L/L genotype may lack the preventive concerns that discourage free-riding, mostly in non-threatening circumstances, namely when punishment is absent.

Examining the 5-HTR1B-rs13212041 variant, we found that individuals with the T/T genotype held more optimistic expectations about others’ punishment behavior, that is, they anticipated less frequent punishment or a greater likelihood of prosocial punishment targeting low contributors (Fig. 1c). Given that the T/T genotype of rs13212041 appears to reduce 5-HTR1B expression by enabling the interaction with a regulatory microRNA (miR-96) and that 5-HTR1B acts as an autoreceptor^32^, we hypothesize that this mechanism may lead to an increased serotonergic transmission via reduced negative feedback on serotonin release. This interpretation is consistent with previous evidence indicating that genetic variants associated with enhanced serotonergic signaling are linked to lower levels of punitive responses to unfair behavior as compared to variants associated with reduced serotonin transmission^23,39^. However, it is important to consider that 5-HTR1B also functions as a heteroreceptor, modulating the release of other neurotransmitters. Specifically, 5-HT1B heteroreceptors are expressed on GABAergic, glutamatergic, and cholinergic neurons, where they inhibit the release of GABA, glutamate, and acetylcholine, and they facilitate dopamine release by suppressing GABAergic interneurons that normally inhibit dopaminergic activity^72^. Therefore, the behavioral effects of the 5-HTR1B-rs13212041 T/T genotype likely extend beyond serotonin signaling alone and may involve broader neuromodulatory dynamics.

Turning to analyze the dopaminergic pathway, we found that carriers of the catechol-O-methyltransferase (COMT) rs4680 A/A genotype held more pessimistic beliefs about others’ contributions in the presence of a punishment option, that is, they expected lower contributions from other participants in the Public Goods Game when punishment was allowed (Fig. 1d). The literature indicates that the Val158Met variant reduces COMT enzyme activity, thereby slowing dopamine inactivation. The Met/Met genotype (A/A) has been associated with dysfunctional cost–benefit evaluations, lower levels of cooperation in group contexts, and reduced scores on cooperation scales^44,45^. Moreover, in a study where participants were asked to donate part of a previously earned endowment to a child from a developing country, A/A homozygotes donated approximately half as much as G-allele carriers^73^. This behavior may reflect selfish tendencies, a miscalculation of the costs and benefits associated with punishment, or a stronger sense of entitlement to personal earnings. The latter interpretation aligns with Goldman’s “warrior/worrier” hypothesis, which posits that the Val allele is linked to a greater stress resilience (“warrior”), while the Met allele is associated with increased anxiety and cognitive sensitivity (“worrier”)^74^. These findings, along with studies linking the Met allele to heightened negative emotionality^75^, may help explain why A/A carriers had lower expectations of fair contributions from others—even in a context where punishment is possible.

Some limitations of this study should be acknowledged. The sample of participants and the selection of dopaminergic and serotonergic gene variants were drawn from a pre-existing and ongoing project at the University of Pisa, which originally had different research objectives. The subset of participants who agreed to take part in the current experiment was relatively small for a genetic study, and the number of genotyped variants—although focused on key genes implicated in social behavior—may be considered limited for a comprehensive investigation of this topic. Future research should aim to include a larger number of participants, a broader range of genetic polymorphisms, a variety of economic games, and more refined measures of individual beliefs. Additionally, due to limited statistical power or due to the possible differences in the prosocial construct highlighted by the Public Goods Game, we were unable to replicate previous findings bridging the DRD4 receptor and fairness and altruism in Dictator and Ultimatum games^52–54,56^.

Nonetheless, our results suggest that specific genetic variants from both serotonergic and dopaminergic systems may influence distinct aspects of social decision-making processes. Notably, this study demonstrates that such genetic variants are associated not only with behavior but also with individuals’ expectations of the behavior of others. Overall, these findings contribute to a deeper understanding of the genetic underpinnings of social behavior and underscore the importance of further research into the biological foundations of prosociality and antisociality.

## Methods

### Participants and experimental sessions

Participants were recruited from a larger sample involved in an ongoing project at the University of Pisa, investigating the neurobiological correlates of human social behavior, for which DNA extraction and genotyping had been performed. The study complied with the Declaration of Helsinki and received approval from the University of Pisa Ethics Committee. A total of 104 participants (71 females) agreed to take part in this experiment, conducted online via the Prolific platform using an interface coded in oTree^76^. However, analyses included only 99 participants (67 females; mean age = 32.9 years, SD = 12.3) due to incomplete data from five subjects. All participants had no history of psychiatric or behavioral disorders. Gender showed no significant correlation with genetic variants, behavior, or beliefs in the Public Goods Game. Thanks to the strategy method design^77^, sessions were scheduled flexibly, and group matching was done ex-post. Eight sessions were held during May and June 2020. Participants received a €1.50 show-up fee, with potential earnings up to €6 based on performance. An additional €0.40 bonus was awarded for sufficiently accurate belief predictions (see below), ensuring incentive compatibility of the beliefs elicitation method while providing smaller incentives for beliefs to avoid hedging behavior. All participants provided informed consent online and could withdraw at any time.

### Public Goods Game

In a standard Public Goods Game (PGG), each participant receives an initial endowment, which can be kept or contributed to a common pool that benefits the whole group. The total contributions are multiplied by a constant greater than one and then evenly redistributed. This structure creates an incentive to free ride, keeping one’s endowment while benefiting from others’ cooperation, although the socially optimal outcome is achieved when all contribute fully. A punishment option can be introduced, allowing players to reduce others’ payoffs at a personal cost. This mechanism is typically used to discourage defection and promote cooperation^3^. Real-world analogs include tax compliance, climate agreements, or shared resource management—for example, tax evaders still benefit from public services funded by others. In our experiment, participants played two versions of a one-shot, anonymous PGG in groups of four. Each received 10 points to either keep or contribute. The total pool was doubled and equally divided. The Nash equilibrium was to contribute nothing, while the social optimum was full contribution (yielding 20 points per player). In the punishment version, after the contribution stage, participants could spend 2 points to deduct 4 points from another player’s total. No feedback was given, following the use of the strategy method^77^. Punishment options included: punishing the lowest contributor (prosocial), the highest contributor (antisocial), no punishment (second-order free-riding), or punishing randomly (spiteful). Belief elicitation rewarded accuracy. Participants earned a bonus for guessing the average contribution within one point and for identifying both the most and least common punishment types.

### DNA extraction and genotyping

Saliva samples were collected using Oragene tubes (DNA Genotek Inc., Ottawa, Canada), and DNA was extracted with the prepITL2P kit following the manufacturer’s instructions. We genotyped four dopaminergic variants (ANKK1-rs1800497, SLC6A3 40 bp VNTR, DRD4 48 bp exon III VNTR, COMT-rs4680) and four serotonergic polymorphisms (TPH2-rs4570625, 5-HTR1B-rs13212041, 5-HTR2A-rs6314, and 5-HTTLPR, including rs25531). For HTR1B-rs13212041, HTR2A-rs6314, TPH2- rs4570625, and ANKK1-rs1800497, homozygotes for the minor allele were grouped with heterozygotes and compared to homozygotes for the ancestral allele, a common approach in genetic studies to reduce the degrees of freedom and thus increase the statistical power of the analyses, which is particularly useful when sample size is small (i.e., HTR1B-rs13212041 C/C + C/T vs. T/T; HTR2A-rs6314 C/C + C/T vs. T/T; TPH2-rs4570625 T/T + G/T vs. G/G; ANKK1-rs1800497 C/C + C/T vs. T/T; see^78^). For COMT-rs4680, genotypic groups were defined based on functional data^79^. Specifically, the low-activity genotype (A/A) was compared to the high and intermediate-activity genotypes (G/G and G/A). The same strategy was applied to VNTR polymorphisms: DRD4 exon III VNTR and 5-HTTLPR, which includes the functional SNP rs25531 (A > G; rs25531, NCBI ID: 16,642,437). In particular, DRD4 low-activity alleles (non-4r) were compared to the high-activity genotype (4r/4r)^80^, while for 5-HTTLPR, low-activity genotypes (S/S, S/L_G_, S/L_A_, and L_G_/L_A_) were compared to the high-activity genotype (L_A_/L_A_). The S (short) and L (long) alleles are the most common variants of 5-HTTLPR. Evidence suggests that the S allele reduces 5-HTT expression^21,22^, and the L_G_ variant (the L allele with the G nucleotide at rs25531) has a similar effect^81^. Although the G nucleotide is less frequent in the S allele, it does not further decrease 5-HTT transcription compared to the S_A_ allele^82^. For SLC6A3-VNTR, non-9r alleles were compared to the 9r/9r genotype^70^. Except for TPH2-rs4570625 and SLC6A3 40 bp VNTR, all the genetic variants are in Hardy–Weinberg equilibrium (Table 3), a principle for which the genetic variation in a population will be constant across generations, in the absence of disturbing factors^83^. When studying genes of a restricted sample (such as in the case of an experiment), H-W equilibrium is used to state that the distribution of allele variation in the sample is not different from the one of the general population to avoid hyper- or hypo-representation of some genetic variants, hence resulting in misleading results. In our case, although TPH2-rs4570625 and SLC6A3 40 bp VNTR were kept in the analysis, their (non-significant in any case) results were not considered since these two genes were, in our sample, not distributed according to H-W equilibrium.

**Table 3:** Hardy–Weinberg Equilibria of the genotyped polimorphisms.

	Genotype groupings	Genotypes	Whole sample (n = 104)
HTR1B-rs13212041	T/T	T/T	0.75
	C-allele	C/C	0.019
		C/T	0.231
	H-W eq	*p* = 0.922	
5HTTPLR-rs25531	L/L	L/L	0.231
	S/S	S/S	0.269
		S/L	0.50
	H-W eq	*p* = 0.987	
HTR2A-rs6314	T/T	T/T	0.009
	C-allele	C/C	0.778
		C/T	0.211
	H-W eq	*p* = 0.711	
TPH2-rs4570625	T/T	T/T	0.019
	G-allele	G/G	0.538
		G/T	0.442
	H-W eq	*p* = 0.03	
SLC6A3 40 bp VNTR	9r/9r	9r/9r	0.096
	non-9r allele	9r/non-9r	0.548
		non-9r/non-9r	0.355
	H-W eq	*p* = 0.073	
ANKK1-rs1800497	T/T	T/T	0.009
	C-allele	C/C	0.721
		C/T	0.269
	H-W eq	*p* = 0.355	
DRD4 48 bp exon III VNTR	4r/4r	4r/4r	0.413
	non-4r allele	4r/non-4r	0.480
		non-4r/non-4r	0.105
	H-W eq	*p* = 0.526	
COMT-rs4680	A/A (Met/Met)	A/A	0.211
	G-allele	G/G	0.336
		A/G	0.451
	H-W eq	*p* = 0.404	

### Data analysis

Eight genetic variants and six behavioral variables were correlated, resulting in 48 tests. Spearman correlations were used to account for the presence of non-normalities in the data. Table 1 shows the correlation coefficients with their respective uncorrected *p*-values, Pu. Subsequently, a standard Bonferroni correction^84^ of a factor of 48 was applied to control for family-wise error rate (FWE) therefore correcting p-values for multiple testing (Pc). This correction returned four correlation coefficients (three for the serotonergic pathway and one for the dopaminergic pathway) that remained statistically significant. We confirmed our exploratory correlations with non-parametric tests (Wilcoxon-Mann–Whitney and Pearson’s chi-square test). Size effects were computed using Glass’ Δ, while marginal effects and the coefficient of determination of our results were computed using linear regression models.

All the computations were performed with Stata 16. Specifically, for the PGG, we considered the subject’s contribution to the joint project, PGG_C_, for the Public Goods Game without punishment, and PGG_P_, for the version with the punishment option. Similarly, we considered the associated beliefs of the actions in the two games, bPGG_C_ and bPGG_P_, respectively. Considering the punishment itself, we created a dummy variable, *ANTI*, in which we merged the antisocial and spiteful punishment choices (with value 1), as opposed to the prosocial punishment and the absence of it (with value 0). Then, since the beliefs about punishment were stated as the punishment action that people believed to be more common, and not as a proportion, another dummy variable was created*, bmANTI*. In this case, 1 was given if the subject believed the more common punishment behavior was antisocial or spiteful, and 0 was given otherwise. Lastly, based on previous literature indicating functional associations between certain alleles and prosocial or antisocial behavior (as detailed in the Introduction), genotypes were grouped depending on the allele of interest, coding them into dummy variables. Specifically, genotypes previously associated with prosocial behavior were coded as 1 for 5-HTR2A (T/T), DRD4 (4r/4r), TPH2 (T/T), and SLC6A3 (9r/9r), and 0 otherwise. Conversely, genotypes associated with antisocial, impulsive, or risky behaviors were coded as 1 for 5-HTTLPR (L/L), COMT (Met/Met), HTR1B (T/T), and ANKK1 (T/T), and 0 otherwise.

## Supplementary Information

Supplementary Information.

## Data Availability

Data and Supplementary Materials are provided into a online repository. Data: https://doi.org/10.6084/m9.figshare.26090566.v1 Materials: https://doi.org/10.6084/m9.figshare.26090563.

## Materials availability

Materials are available in our online repository.
