# Supplementary material for: Dopaminergic and serotonergic genetic variants predict actions and expectations of cooperation and punishment
Source: Sci Rep. 2025 Jul 1;15:20641. doi: 10.1038/s41598-025-03772-4 (PMC12217337; doi:10.1038/s41598-025-03772-4)
Supplement: Supplementary file 1 — Supplementary Information. [file 41598_2025_3772_MOESM1_ESM.pdf]

Supplementary Information for “Dopaminergic and serotonergic genetic variants predict actions and expectations of cooperation and punishment”

Pablo Marcos-Prieto[1,†,*], Erica Ordali[1,†,*], Veronica Mariotti[2], Sara Palumbo[2], Stefano Vellucci[2], Emiliano Ricciardi[1], Leonardo Boncinelli[3], Pietro Pietrini[1], Silvia Pellegrini[3,†], and Ennio Bilancini[1,†]

[1]IMT School for Advanced Studies Lucca
[2]Department of Clinical and Experimental Medicine, University of Pisa
[3]Department of Economics and Management, University of Florence

[*]corresponding authors: Pablo Marcos-Prieto, Erica Ordali, (pablo.prieto@imtlucca.it, erica.ordali@imtlucca.it)
[†]these authors contributed equally to this work

May 13, 2025

Disclaimer

The following Supplementary Information provides additional results and materials for the procedures described in the paper “Dopaminergic and serotonergic genetic variants predict actions and expectations of cooperation and punishment”. We report the results for all the variables for each genetic variant, the instructions of the experiment, and the list of variables. However, the instructions of the experiment described here refer to a larger experiment, of which we describe here only the games used in the aforementioned paper.

A Additional Results

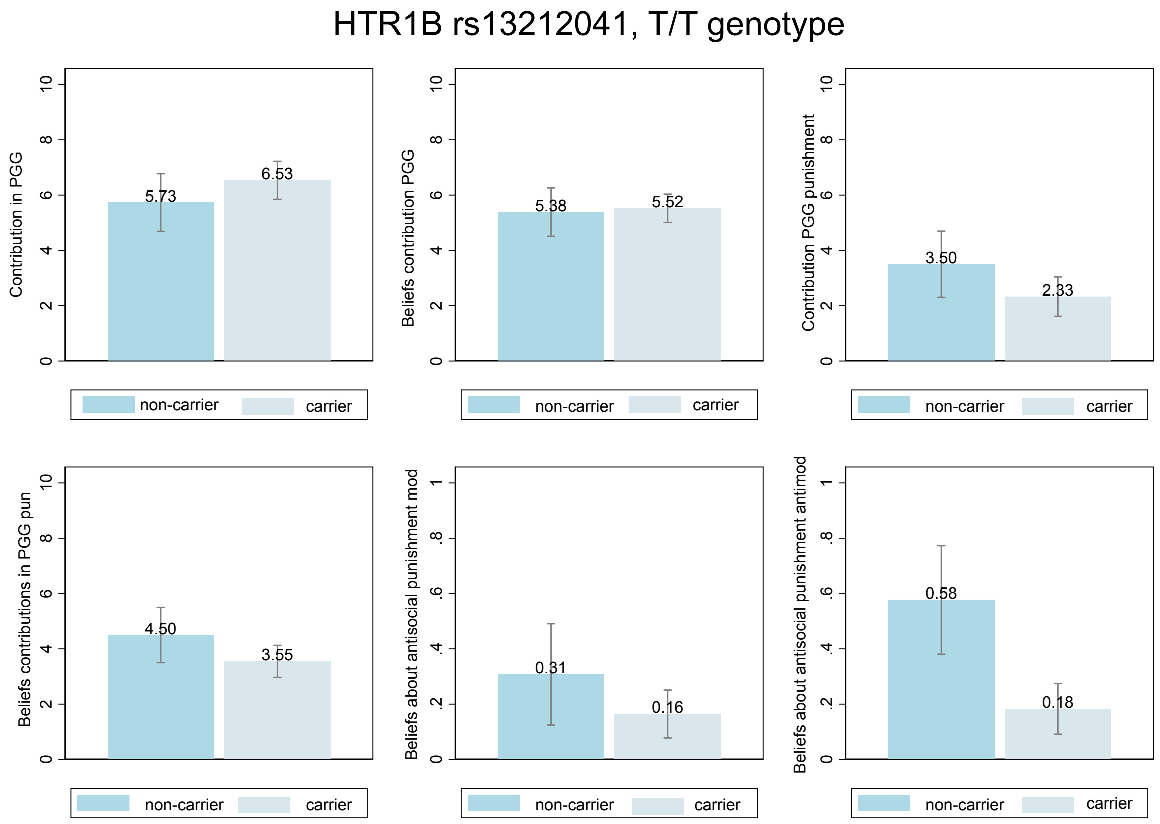

Figure 1: Plot representation of the results for the T/T genotype of HTR1B. Panels show the average behaviors and beliefs of participants (non-carriers vs. carriers) in the PGG with and without punishment. Bars represent standard errors.

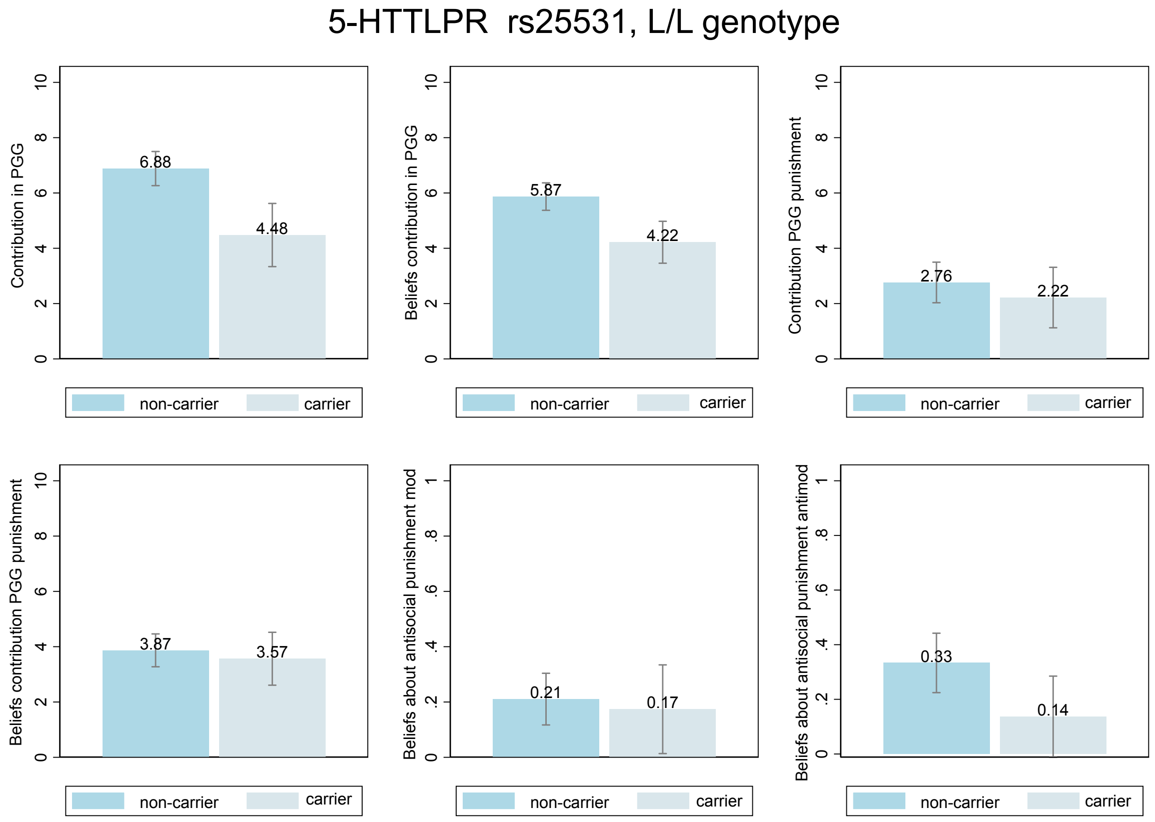

Figure 2: Plot representation of the results for the L/L genotype of 5-HTTLPR. Panels show the average behaviors and beliefs of participants (non-carriers vs. carriers) in the PGG with and without punishment. Bars represent standard errors.

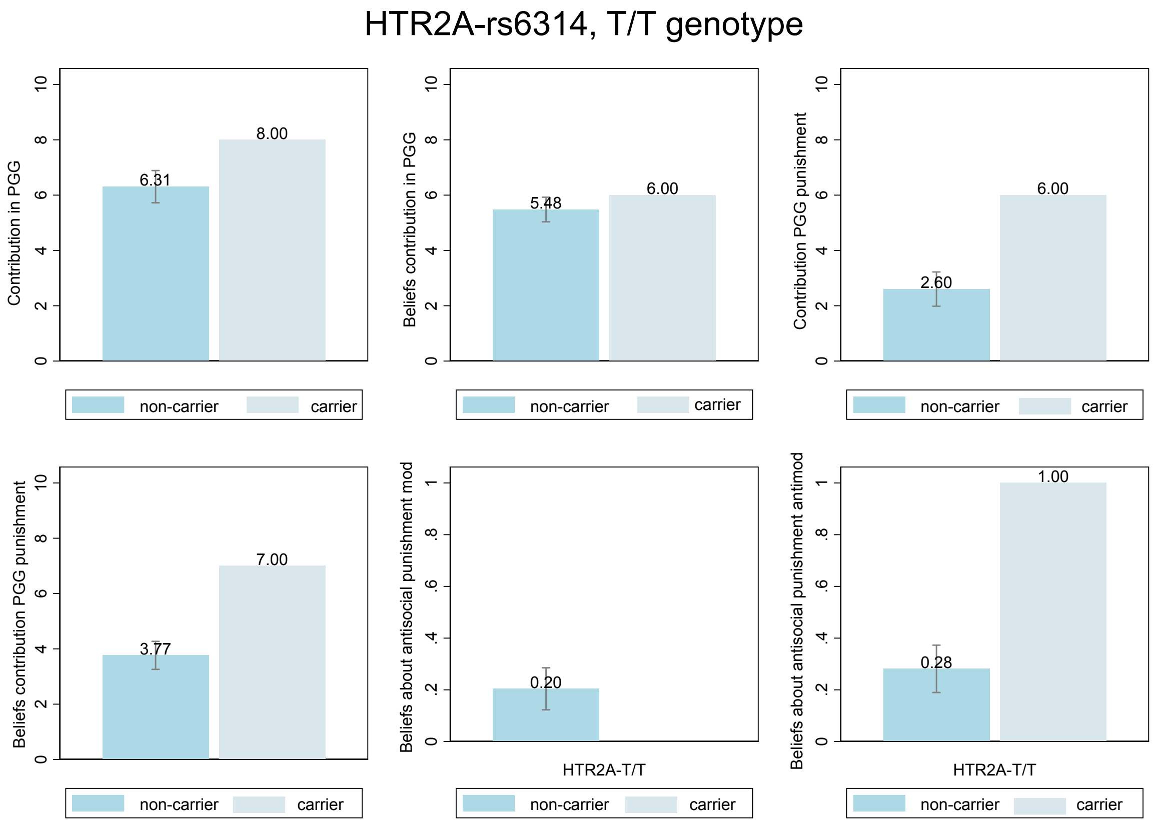

Figure 3: Plot representation of the results for the T/T genotype of 5-HTR2A. Panels show the average behaviors and beliefs of participants (non-carriers vs. carriers) in the PGG with and without punishment. Bars represent standard errors.

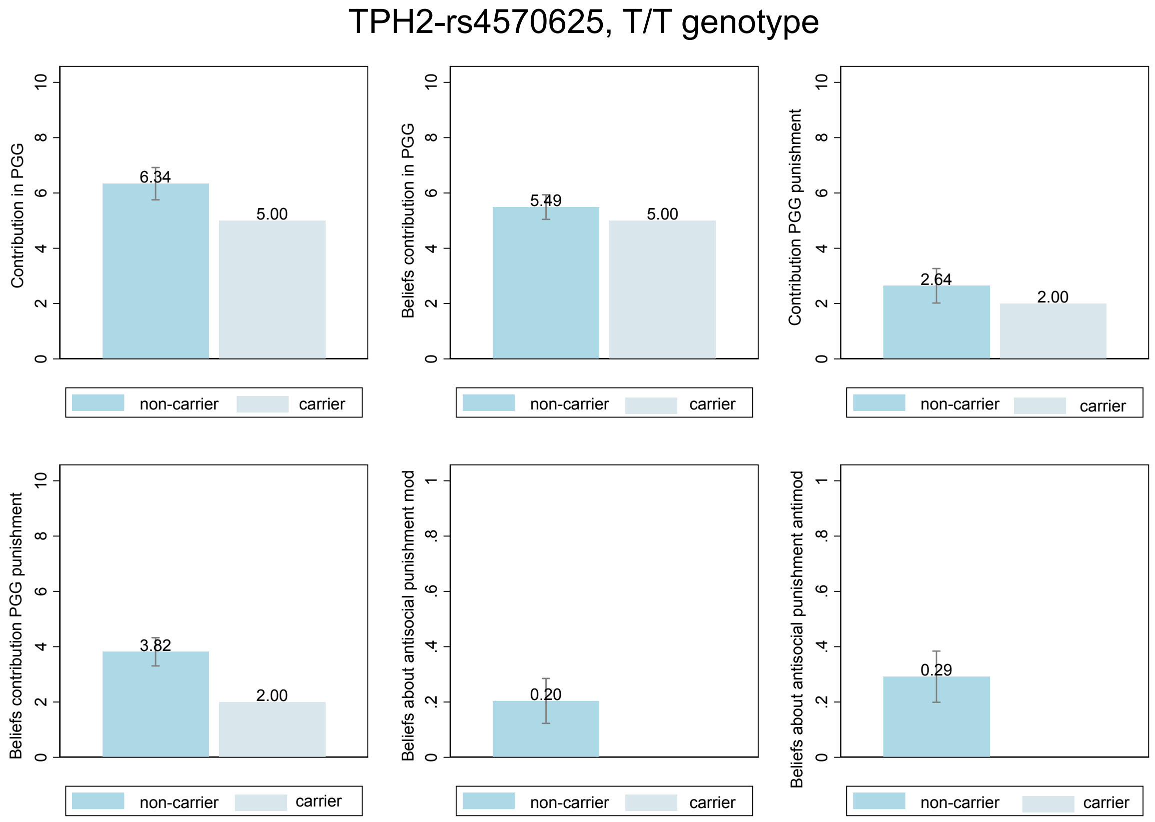

Figure 4: Plot representation of the results for the T/T genotype of TPH2. Panels show the average behaviors and beliefs of participants (non-carriers vs. carriers) in the PGG with and without punishment. Bars represent standard errors.

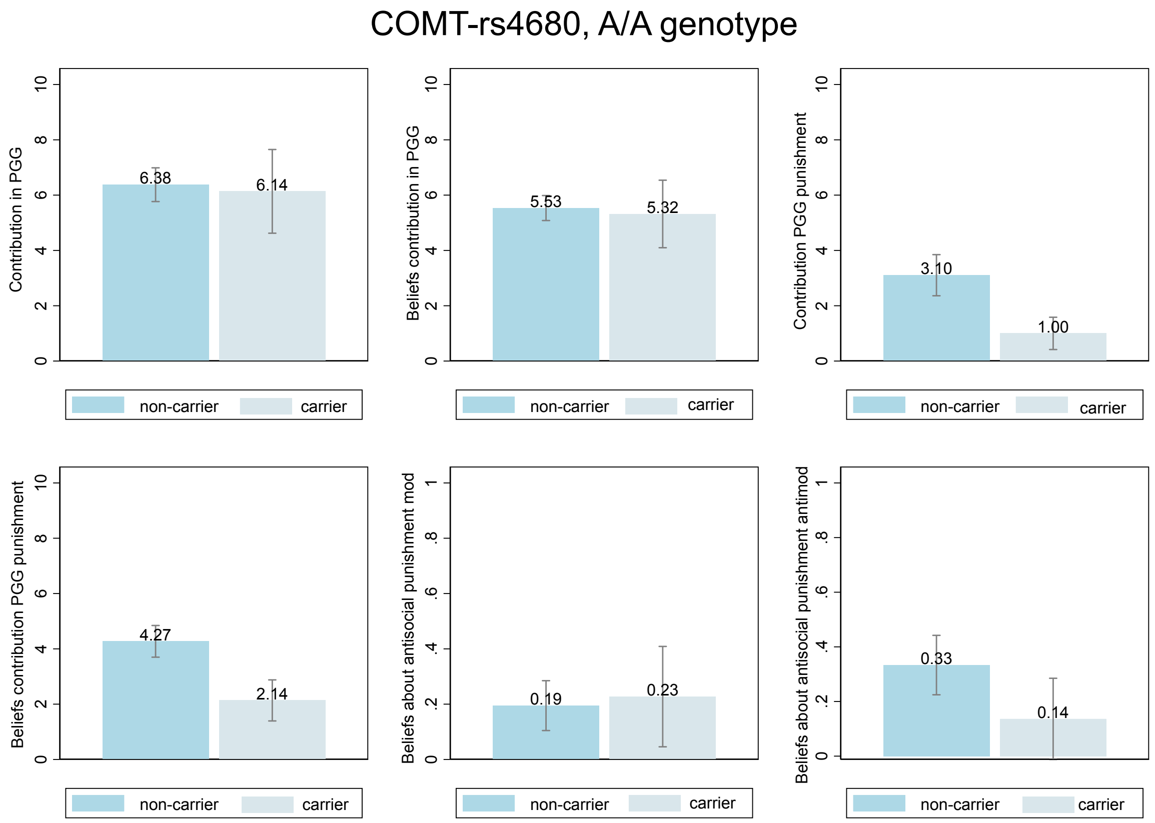

Figure 5: Plot representation of the results for the A/A genotype of COMT. Panels show the average behaviors and beliefs of participants (non-carriers vs. carriers) in the PGG with and without punishment. Bars represent standard errors.

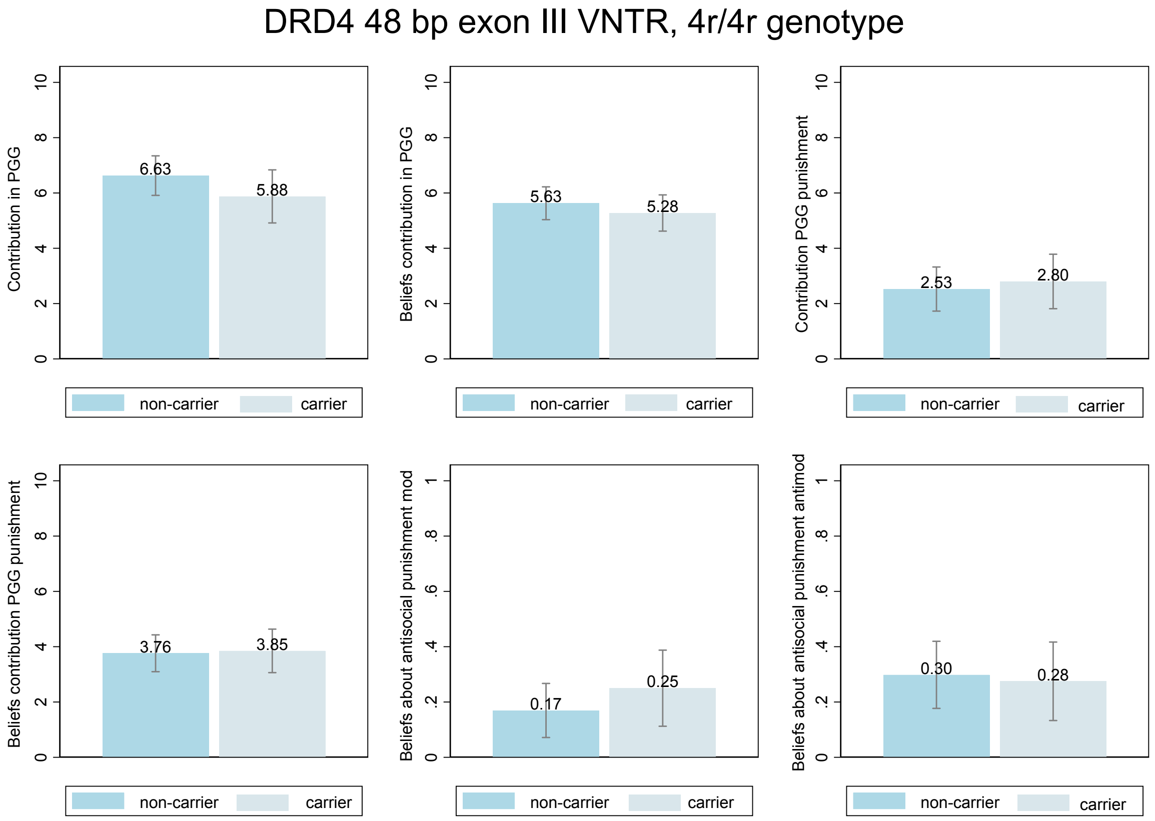

Figure 6: Plot representation of the results for the 4r/4r genotype of DRD4. Panels show the average behaviors and beliefs of participants (non-carriers vs. carriers) in the PGG with and without punishment. Bars represent standard errors.

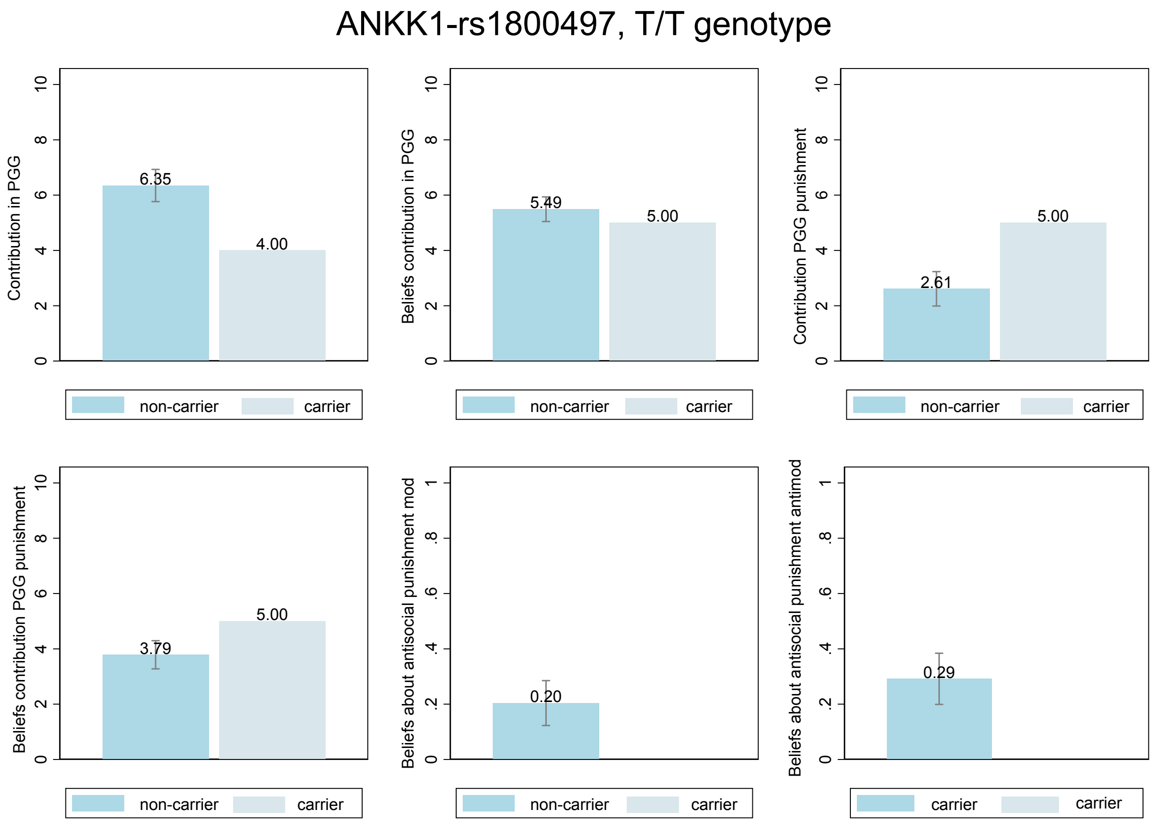

Figure 7: Plot representation of the results for the T/T genotype of ANKK1. Panels show the average behaviors and beliefs of participants (non-carriers vs. carriers) in the PGG with and without punishment. Bars represent standard errors.

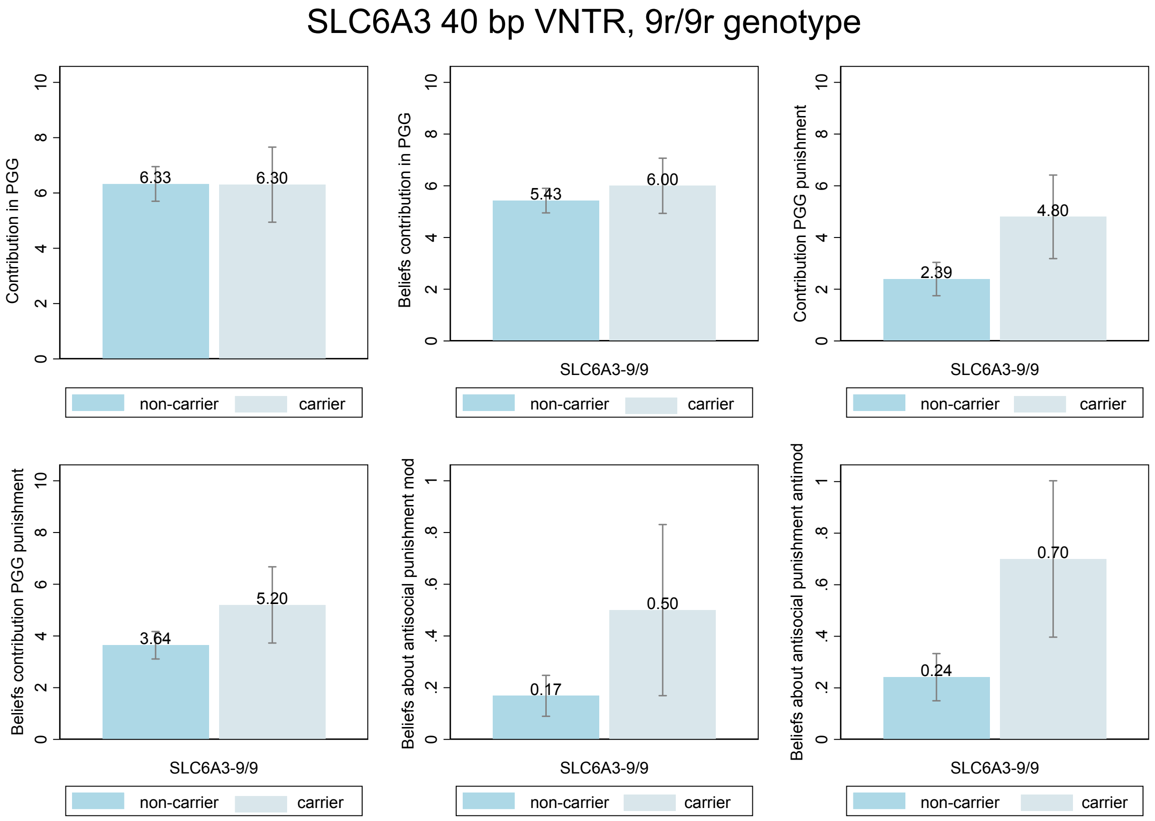

Figure 8: Plot representation of the results for the 9r/9r genotype of SLC6A3. Panels show the average behaviors and beliefs of participants (non-carriers vs. carriers) in the PGG with and without punishment. Bars represent standard errors.

B Instructions of the experiment (original Italian)

B.1 Instructions

Benvenuto!

In questo esperimento ti sarà chiesto di compiere delle scelte.
L'esperimento avrà una durata di circa 15 min.

Le scelte che dovrai compiere saranno relative ad una serie di situazioni in cui sarai abbinato a caso con un altro partecipante a questo esperimento.
Per ogni nuova situazione sarai abbinato con un **nuovo partecipante**.
Non avrai modo di conoscere l'identità dell'altro partecipante e **non interagirai mai con lo stesso** partecipante più di una di una volta.

In ogni situazione le conseguenze delle scelte compiute da te e dall'altro partecipante determineranno un ammontare di punti che tu e l'altro partecipante guadagnerete.
Alla fine dell'esperimento sarai pagato in base ai punti ottenuti.
Nello specifico, in ogni situazione ti sarà chiesto di compiere delle scelte in condizioni diverse e formulare delle ipotesi.
Alla fine dell'esperimento, **una tra le scelte e una tra le ipotesi saranno estratte a caso** e l'ammontare dei punti guadagnati in quelle occasioni andrà ad **incrementare** la tua ricompensa di base, con un tasso di conversione pari a 1 euro ogni 20 punti.

Ogni situazione é differente, per cui leggi attentamente le istruzioni che troverai man mano.
Per continuare, insersci il tuo codice partecipante e poi clicca sul pulsante *"Successivo"*.

Codice partecipante:

Successivo

B.2 Public Goods Game without punishment

Descrizione della situazione

In questa situazione, tu sei abbinato a caso con **tre partecipanti** all'esperimento.
Tu e gli altri partecipanti avete una **base di partenza di 10 punti**.
Tu e ciascuno degli altri partecipanti potete contribuire **con una parte** della propria base di punti.

La somma dei punti che tu e gli altri tre partecipanti avete contribuito verrà moltiplicata per due e poi divisa tra voi quattro, indipendentemente da quanto ciascuno di voi ha contribuito.
I punti che otterrai saranno dati da questa divisione più la parte dei punti della tua base che non hai utilizzato.

Quanti punti contribuisci?

○ 0 ○ 1 ○ 2 ○ 3 ○ 4 ○ 5 ○ 6 ○ 7 ○ 8 ○ 9 ○ 10

Successivo

Formula un'ipotesi

Nella situazione precedente hai deciso con quanti punti, della tua base di partenza, contribuire. Secondo te, con quanti punti **in media** i partecipanti a questo esperimento hanno contribuito?

Inserisci un numero intero tra 0 e 10:

La tua ipotesi sarà considerata corretta se avrà uno scarto non superiore ad un punto rispetto alla media effettiva.
La risposta corretta ti dà un **bonus di 2 punti**.

Successivo

B.3 Public Goods Game with punishment

Descrizione della situazione

In questa situazione, tu sei abbinato a caso con altri **tre partecipanti** all'esperimento.
Tu e gli altri partecipanti avete una **base di partenza di 10 punti**.
Tu e ciascuno degli altri partecipanti potete contribuire **con una parte** della propria base di punti.

Tu e ciascuno degli tre altri partecipanti potete decidere di applicare una sanzione:
ciascuno di voi quattro può spendere 2 dei propri punti per **ridurre di 4 i punti del partecipante che intende sanzionare**.

Quanti punti contribuisci?

○ 0 ○ 1 ○ 2 ○ 3 ○ 4 ○ 5 ○ 6 ○ 7 ○ 8 ○ 9 ○ 10

Chi indendi sanzionare?

○ Uno a caso degli altri
○ Quello degli altri che ha contribuito di meno
○ Quello degli altri che ha contribuito di più
○ Nessuno degli altri

Successivo

Formula un'ipotesi

Nella situazione precedente hai deciso con quanti punti, della tua base di partenza, contribuire.
Allo stesso tempo hai deciso quale sanzione applicare.

Secondo te, quanti punti **in media** i partecipanti a questo esperimento hanno contribuito?

Inserisci un numero intero tra 0 e 10:

La tua ipotesi sarà considerata corretta se avrà uno scarto non superiore ad un punto rispetto alla media effettiva.
La risposta corretta ti dà un **bonus di 2 punti**.

Secondo te, seconde te qual è l’opzione che è stata scelta di meno?

○ Uno a caso degli altri
○ Quello degli altri che ha contribuito di meno
○ Quello degli altri che ha contribuito di più
○ Nessuno degli altri

Secondo te, seconde te qual è l'opzione che è stata scelta di più?

- ◯ Uno a caso degli altri
- ◯ Quello degli altri che ha contribuito di meno
- ◯ Quello degli altri che ha contribuito di più
- ◯ Nessuno degli altri

La tua ipotesi sarà considerata corretta se avrà uno scarto non superiore ad un punto rispetto alla media effettiva.
La risposta corretta ti dà un **bonus di 2 punti**.

Successivo

C Instructions of the experiment (English translation)

C.1 Instructions

Welcome!

In this experiment you will be required to make some choices.
The experiment will be on average 15 minutes-long.
The choices you will have to make are relative to a series of situations in which you will be randomly paired with another participant in this experiment.
For every new situation, you will be paired with **a new participant.**
You won't know the identity of the other participant, and **you will never interact with the same participant twice.**

In every situation the consequences of the choices made by you and by the other participant will determine an amount of points that you and the other will earn throughout the experiment.
At the end of the experiment, you will be paid according to the earned points.
Specifically, in every situation you will be asked to make some choices in different conditions and to formulate some hypotheses.
At the of the experiment **one between the choices and one between the hypotheses will be extracted randomly** and the amount of points collected in those occasions will **increment** your base reward, with a conversion rate of 1 euro every 20 points.

Every situation is different, so read carefully the instructions that you will find every time.
To continue, insert your participant code and click on the button *"Next"*.

C.2 Public Goods Game without punishment

Situation's description
In this situation you are randomly paired with **three participants** of these experiment. You and the others have a **starting base of points of 10 points each.** You and the others can contribute **with a part** of your starting base.

The sum of points that you and the other participants contributed will be multiplied by two and then divided between you four, independently of the amount of points that each of you actually contributed. The points obtained in the end will derived from the sum of this division plus the points of your base that you did not used.

How many points do you want to contribute?

Make a guess
In the previous situation you decided with how many points of your personal starting-base contribute.

In your opinion, with how many points, **on average**, the participants to this experiment contributed?

Your hypothesis will be considered correct if it will have a difference from the actual percentage not larger than five percentage points. A correct answer will give you 2 **bonus points.**

C.3 Public Goods Game with punishment

Situation's description
In this situation you are randomly paired with **three participants** of this experiment. You and the others have a **starting base of points of 10 points each.** You and the others can contribute **with a part** of your starting base.

You and the other three participants can choose to apply a **sanction**: each of you four can spend 2 of their personal points to **reduce by 4 the points of the participant that you want to sanction.**

How many points do you want to contribute?

Who you want to punish?

- A random person between the others
- The one that contributed less
- The one that contributed more
- No one of the others

Make a guess
In the previous situation you decided with how many points of your personal starting-base contribute. At the same time, you decided which sanction to apply.

In your opinion, with how many points, **on average**, the participants to this experiment contributed?

Your hypothesis will be considered correct if it will have a difference from the actual percentage not larger than one point. A correct answer will give you **2 bonus points**.

In your opinion which is the option that was chosen the most?

- A random person between the others
- The one that contributed less
- The one that contributed more
- No one of the others

In your opinion which is the option that was chosen the less?

- A random person between the others

- The one that contributed less
- The one that contributed more
- No one of the others

Your hypothesis will be considered correct if it will have a difference from the actual percentage not larger than one point. A correct answer will give you **2 bonus points.**

D Legend of variables

D.1 Behavioral variables

D.1.1 Actions

- **PGGc:** contribution in Public Good Game without punishment. Domain in [0,10].
- **PGGcp:** contribution in Public Good Game with punishment. Domain in [0,10].
- **Punishment:** punishment modality. Domain in {*one*, *less*, *more*, *none*}.

D.1.2 Beliefs

- **bPGGc:** belief about average contribution in the Public Good Game withoug punishment. Domain in [0,20].
- **bPGGcp:** belief about average contribution in the Public Good Game withoug punishment. Domain in [0,20].
- **bmodalPunishment:** belief about modal punishment modality. Domain in {*one*, *less*, *more*, *no*[illegible]
- **bantimodalPunishment:** belief about antimodal punishment modality. Domain in {*one*, *less*, *more*, *none*}.

D.2 Genetic variables

D.2.1 Serotonergic pathway

- **HTR1B (rs13212041):** 5-idroxytryptamine receptor 1B. Three genotypes are found here; homozygous for high expression allele (C/C), homozygous for the low expression allele (T/T) or heterozygous alleles (C/T).
- **5HTTPLR-SLC6A4 (rs25531):** 5-idroxytryptamine transporter-linked polymorphic region. This gene possess three genotypes, according to the homozygosity for long alleles (L/L) or short alleles (S/S) or heterozygosity (S/L).
- **HTR2A (rs6314):** 5-idroxytryptamine receptor 2A. Three genotypes are found here; homozygous for high expression allele (C/C), homozygous for the low expression allele (T/T) or heterozygous alleles (C/T).
- **TPH2 (rs4570625):** tryptophan hydroxylase 2. The genotypes are distinguished based on homozygousity for high expression allels (G/G) or for low expression ones (T/T), and heterozygosity (G/T).

D.2.2 Dopaminergic pathway

- **DAT-1-uVNTR:** dopamine transporter, untranslated Variable Number of Tandem Repeat. Most common repetition found in the population are the allelic couplets: 9R/9R, 9R/10R, 10R/10R and 10R/11R.
- **ANKK1 (rs1800497):** ankyrin repeat and kinase domain containing 1. Genotypes for this gene are found based on homozygosity for high expression alleles (C/C), low expression ones (T/T) or heterozygous alleles (C/T).
- **DRD4-VNTR:** dopamine receptor D4 exon III-48 bp Variable Number of Tandem Repeat. Most common couplets of alleles in the population are: 4R/4R, 4R/7R, 2R/4R, 7R/7R, 2R/7R.
- **COMT (rs4680):** Catechol-O-methyltransferase (Val158Met). This gene possess three genotypes, based on the distinction of homozygosity of the low activity protein Met (A/A) and the high activity protein Val (G/G) or heterozygosity (G/A).

D.3 Other variables

D.3.1 Demographics

- **age:** years of age.
- **educ:** years of education.
- **gender:** gender dummy.
- **vol:** dummy for volunteering.

D.3.2 Questionnaires

- **MOPSmi:** MOPS questionnaire, mother indifference. Domain in [0,18].
- **MOPSma:** MOPS questionnaire, mother abuse. Domain in [0,15].
- **MOPSmhc:** MOPS questionnaire, mother hyper-control. Domain in [0,12].
- **MOPSfi:** MOPS questionnaire, father indifference. Domain in [0,18].
- **MOPSfa:** MOPS questionnaire, father abuse. Domain in [0,15].
- **MOPSfhc:** MOPS questionnaire, father hyper-control. Domain in [0,12].
- **BISia:** BIS scale, attentive impulsivity. Domain in [0,35].
- **BISIim:** BIS scale, motor impulsivity. Domain in [0,35].
- **BISIinp:** BIS scale, non-planification impulsivity. Domain in [0,35].
- **BIStot:** BIS scale, total. Domain in [0,105].
